# Supplementary material for: Methylxanthines Modulate Circadian Period Length Independently of the Action of Phosphodiesterase
Source: Microbiol Spectr. 2023 Jun 5;11(4):e03727-22. doi: 10.1128/spectrum.03727-22 (PMC10434132; doi:10.1128/spectrum.03727-22)
Supplement: Supplemental file 1 — Supplemental material. Download spectrum.03727-22-s0001.docx, DOCX file, 7.2 MB [file spectrum.03727-22-s0001.docx]

**Supplementary Information**

Methylxanthines modulate circadian period length independently of the action of phosphodiesterase

Consuelo Olivares-Yañez^1,2^, María P. Alessandri^1,3^ , Loreto Salas^1,3^, Luis F. Larrondo^1,3,^*

1 ANID-Millennium Science Initiative Program – Millennium Institute for Integrative Biology (iBio). Santiago 8331150, Chile; cdolivar@gmail.com (C.O-Y.), mdpalessa@gmail.com (M.P.A), llarrondo@bio.puc.cl (L.F.L.)

2 Centro de Biotecnología Vegetal, Facultad de Ciencias de la Vida, Universidad Andrés Bello, Santiago 8370186, Chile; cdolivar@gmail.com (C.O-Y.)

3 Departamento de Genética Molecular y Microbiología, Facultad de Ciencias Biológicas, Pontificia Universidad Católica de Chile, Santiago 8331150, Chile; mdpalessa@gmail.com (M.P.A), llarrondo@bio.puc.cl (L.F.L.)

* Correspondence: llarrondo@bio.puc.cl (L.F.L.)

**Supplementary Figure S1. Genotyping of *∆pkac1; ∆pkac2* and *Δpkac1/pkac2* strains.**

Mutant strains were genotyped through PCR. A fragment of the ORF of each gene of interest was amplified in a multiplex PCR, confirming also DNA quality by amplifying an unrelated locus (*rco-1*, 1000bp). A specific band for each loci is observed: for *pkac-1* lanes 1,5,9 &13, for *pkac-2* lanes 3, 7, 11 & 15. Correct insertion of each KO cassette was assessed using primers specific for each locus and a *hph* internal primer (oL116). For *pkac-1* (***in1***: insertion of *hph* at the *pkac-1* locus) lanes 2, 6, 10 & 14. For *pkac-2* (***in2***: insertion of *hph* at the *pkac-2* locus) lanes 4, 8, 12 & 16. Double mutant strains were confirmed through PCR, by assessing the loss of both ORFs of interest, and the correct insertion of the cassette (lanes 13 to 16).


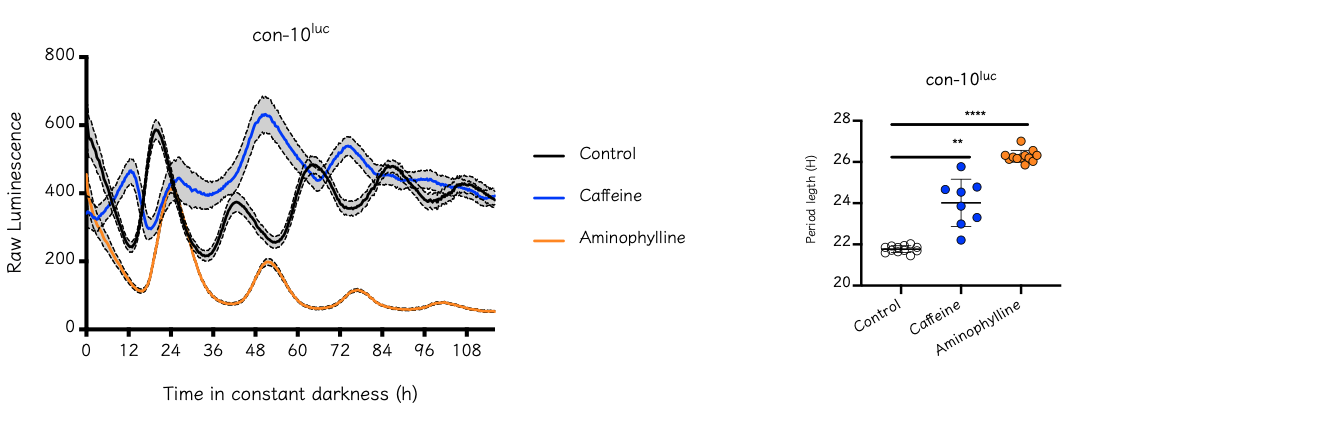


**Supplementary Figure S2**. The addition of methylxanthines increases the period length of the output reporter *con-10*.

The expression of a circadian output *con-10^luc^* translational reporter was evaluated under treatment with two methylxanthines: Caffeine and Aminophylline. The drug treatments increase period length of this reporter. Period determination was performed using Biodare 2.0 (time window of 16-116h) and FFT-NLLS algorithm. Linear Det. Period ± SD. Kruskal-Wallis test followed by a Dunn's multiple comparison test were performed to evaluate differences. ** p<0.01, **** p<0.0001.

Control: 21.77 ± 0.17, Caffeine 3mM: 24.01 ± 1.15, Aminophylline 3mM: 26.28 ± 0.29.

**Supplementary Figure S3.** The absence of *acon-2* leads to impairment of conidia formation.

Neurospora was grown in solid VM media for seven days in LL conditions. The development of aerial hyphae and conidia was observed in WT and *∆pde-1* strains. In the absence of *acon-2*, only aerial hyphae development is observed; no conidia formation is detected.

**Supplementary Figure S4.**The deletion of PDE-1 does not significantly impact the expression of the central clock reporter *frq_c-box_-luc*.

The expression clock reporter *frq_c-box_-luc* was evaluated in a *∆pde-1* strain. No significant differences in expression of the reporter between both strains were observed.

Period determination was performed using Biodare 2.0 and FFT-NLLS algorithm. Linear Det. Period ± SD. Mann Whitney test was performed to evaluate differences. No significant difference with p<0.05 was found.

WT: 22.12 ± 0.45, *∆pde-1*: 21.85 ± 0.58.

**Supplementary Figure S5.**The addition of methylxanthines increases the period length of the output reporter *con-10^luc^* even in the absence of ACON-2.

The expression of the translational circadian output *con-10* was evaluated under treatment with Caffeine and Aminophylline in a *∆acon-2* strain. Period determination was performed using Biodare 2.0 and FFT-NLLS algorithm. Linear Det. Period ± SD. Kruskal-Wallis test followed by a Dunn's multiple comparison test was performed to evaluate differences. *** p=0.0002, **** p<0.0001.

Control: 21.58 ± 0.39, Caffeine 3mM: 22.96 ± 0.25, Aminophylline 3mM: 24.93 ± 0.87.

**Supplementary Figure S6.**The addition of IBMX severely affects the expression of the clock reporters in *∆acon-2* strain.

IBMX was added to the culture media, at 3 and 4mM, to analyze its effect on the clock in *∆acon-2*. The addition of this drug causes a severe impact in the expression of both analyzed reporters and, for *frq_c-box_-luc*, luciferase activity is minimal, and circadian parameters cannot be faithfully extracted from the data. In the case of *frq^luc^* at 4mM, a similar problem is observed. However, at a lower IBMX dose, we can still observe rhythms with a shorter period than the control condition.

Period determination was performed using Biodare 2.0 and FFT-NLLS algorithm. Linear Det. Period ± SD. Mann Whitney test was performed to evaluate differences. **** p<0.0001.

*frq^luc^* Control: 24.5 ± 0.8, IBMX 3mM: 20.72 ± 0.44.

**Supplementary Figure S7.** The use of cAMP analogs does not impact circadian period length.

The effect of the addition of 8Br-cAMP (A) and Bt2-cAMP (B) in circadian period length was evaluated using the clock reporters *frq_c-box_-luc* and *frq^luc^*. Neither of them generates a significant change on circadian period length. Prior to luciferase measurements, plates were inoculated and incubated for 24 hours in LL conditions.

Period determination was performed using Biodare 2.0 and FFT-NLLS algorithm. Linear Det. Period ± SD. Kruskal-Wallis test followed by a Dunn's multiple comparison test were performed to evaluate differences.

WT *frq_c-box_-luc*: Control: 23.70 ± 0.32, 8Br-cAMP 0.5mM: 23.68 ± 0.79, 8Br-cAMP 1mM: 23.36 ± 0.92.

WT *frq^luc^*: Control: 24.80 ± 0.58, 8Br-cAMP 0.5mM: 24.20 ± 0.59. 8Br-cAMP 1mM: 24.81 ± 0.59.

WT *frq_c-box_-luc*: Control: 23.07 ± 0.22, Bt2-cAMP 1mM: 22.63 ± 1.28, Bt2-cAMP 2mM: 23.27 ± 1.21.

WT *frq^luc^*: Control: 23.61 ± 1.08, Bt2-cAMP 1mM: 23.38 ± 0.60, Bt2-cAMP 2mM: 24.22 ± 0.97.


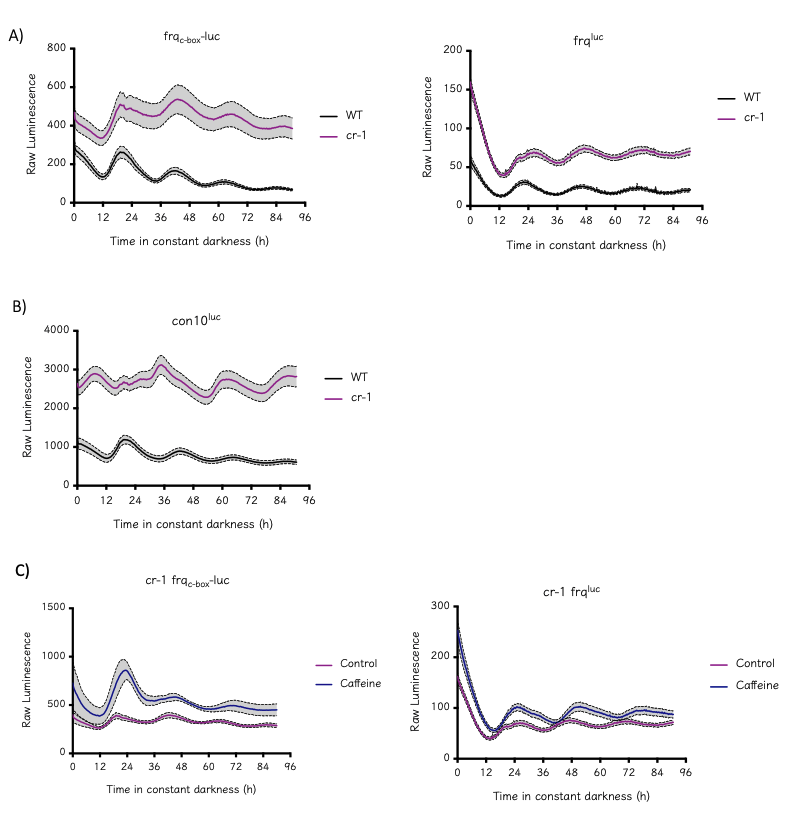


**Supplementary Figure S8.** Circadian rhythms remain normal in the AC mutant *cr-1*.

The state of the central oscillator was evaluated in the historical *cr-1* mutant, which exhibits severely compromised AC activity levels. A) Central clock reporters *frq_c-box_-luc* and *frq^luc^* present clear oscillation with a period close to a WT. A slight increase in period length was observed for the *frq_c-box_luc* reporter. Period determination was performed using Biodare 2.0 and FFT-NLLS algorithm. Linear Det. Period ± SD. Mann Whitney test was performed to evaluate differences. * p=0.0148. *frq_c-box_-luc*: WT: 21.35 ± 0.48, *cr-1*: 22.2 ± 0.72 *. *frq^luc^*: WT: 22.99 ± 0.35, *cr-1* 23.13 ± 0.38.

B) The pattern of the output reporter *con10^luc^* is altered in *cr-1*. Despite the presence of clear oscillations at later times, the high expression and irregular pattern up to 36 hours did not allow proper period determination. It is worth noticing the elevated levels of *con10^luc^* expression in this mutant.

C) Caffeine lengthens period in *cr-1*. Period determination was performed using Biodare 2.0 and FFT-NLLS algorithm. Linear Det. Period ± SD. Mann Whitney test was performed to evaluate differences. Both central reporter present an increase in circadian period length. * p=0.0238. *** p=0.0001. *cr-1 frq_c-box_-luc* Control: 21.96 ± 0.78; Caffeine: 24.42 ± 0.46*. *cr-1 frq^luc^* Control: 23.09 ± 0.37; Caffeine: 26.75 ±0.27***.

**Supplementary Figure S9.** cAMP levels broadly change in mutants of the cognate signaling pathway and upon drug treatment

cAMP levels were measured after 48 hours of growth in LNN-CCD (the media used for luciferase monitoring) for WT, *∆acon-2* and *cr-1* strains. Analyses also included growth in the presence of 3 mM Caffeine or 500 μM 8-Br-cAMP (for WT). In control conditions levels of cAMP are higher in the *∆acon-2* mutant, and almost undetectable in *cr-1,* compared to WT strain. An increase of cAMP is observed in WT when grown in the presence of Caffeine, or the cAMP analog 8-Br-cAMP. No increase of cAMP levels with Caffeine treatment was observed in *∆acon-2*.


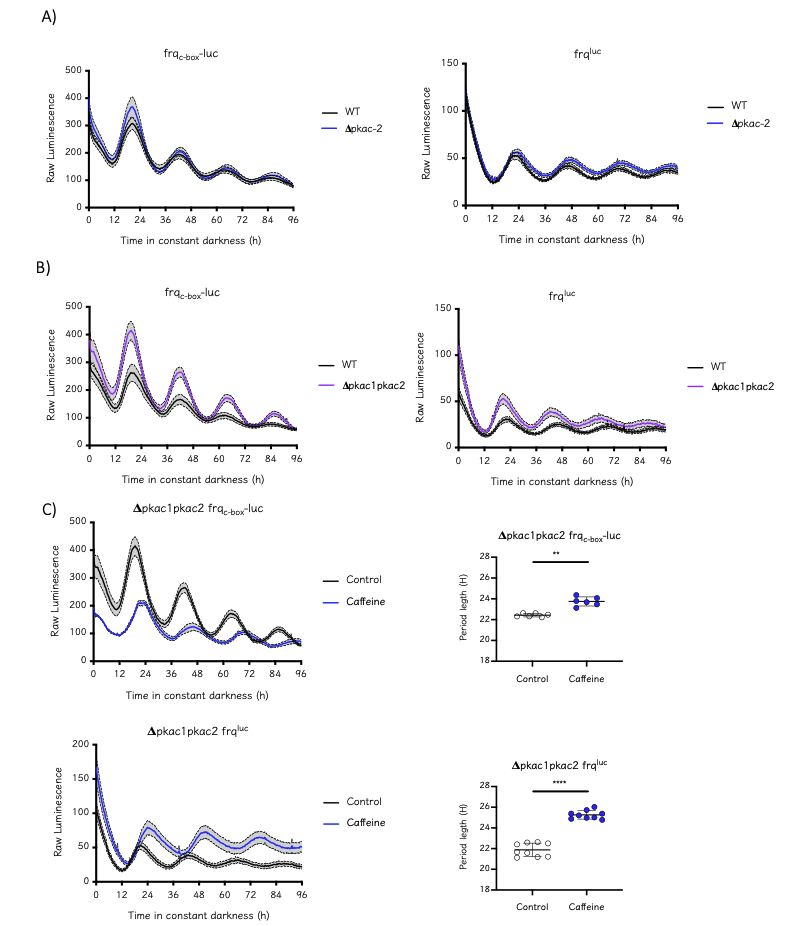


**Supplementary Figure S10. The state of the central oscillator was evaluated in *∆pkac-2* and the *∆pkac1pkac2* double mutant.**

The expression of clock reporters was evaluated in the absence of *pkac-2* and the double mutant *pkac1pkac2.*

A) The absence of *pkac2* does not affect the oscillations of clock reporters.

B) Circadian rhythms are still observed in the *∆pkac1pkac2* strain.

C) Caffeine still exerts period lengthening in the *∆pkac1pkac2* double mutant. A clear increase in period was observed when Caffeine is added to the culture media. Period determination was performed using Biodare 2.0 and FFT-NLLS algorithm. Linear Det. Period ± SD. Mann Whitney test was performed to evaluate differences. *frq_c-box_luc.* Control: 22.43 ± 0.17, *Caffeine:* 23.75 ± 0.43. *frq^luc^.* Control: 21.88 ± 0.64, *Caffeine:* 24.78 ± 0.41.

**Supplementary Figure S11.** Rhythmic expression of the output reporter *con-10^luc^* in the absence of PKAC-1.

A) The expression of *con-10^luc^*, an output reporter, is still rhythmic in the absence of PKAC-1 and in the *∆acon-2* strain, exhibiting a slightly shorter period in the absence of this kinase (B). Period determination was performed using Biodare 2.0 and FFT-NLLS algorithm. Linear Det. Period ± SD Kruskal-Wallis test followed by Dunn's multiple comparison test were performed to evaluate differences. WT *con-10^luc^*: 21.65 ± 0.33, *∆pkac-1 con-10^luc^*:20.74 ± 0.26, *∆acon-2 con-10^luc^*:21.58 ± 0.39

C) High levels of *con-10^luc^* expression are observed in the absence of PKAC-1 (approx. 6.5 more, if the means are compared).
